# Proactive Breakthrough or Passive Exhaustion? A Dual-Path Integrated Model Driven by Perceived Overqualification

**DOI:** 10.3390/bs15050702

**Published:** 2025-05-19

**Authors:** Chuanhao Fan, Bingbing Shang

**Affiliations:** School of Business, Hohai University, Nanjing 211100, China; shangbingbing@hhu.edu.cn

**Keywords:** perceived overqualification, job crafting, role breadth self-efficacy, emotional exhaustion, idiosyncratic deals, cognitive-affective personality system

## Abstract

With the advancement of global economic restructuring and China’s economic transformation, structural employment contradictions have intensified amid increasingly competitive labor markets. The frequent occurrences of “degree devaluation” and talent “downskilling” have made perceived overqualification increasingly prevalent in organizations. This study, based on the Cognitive–Affective Personality System theory, investigates the differential mechanisms through which perceived overqualification drives approach and avoidance job crafting via cognitive and affective pathways. Data from a two-wave survey of 556 Chinese employees produced several key findings: (1) Perceived overqualification significantly enhances approach job crafting while suppressing avoidance job crafting by elevating role breadth self-efficacy (cognitive pathway), demonstrating a proactive breakthrough effect. (2) Perceived overqualification inhibits approach job crafting and exacerbates avoidance job crafting through triggering emotional exhaustion (affective pathway), revealing a passive exhaustion trap. (3) Perceived overqualification exerts a positive and significant overall indirect effect on approach job crafting through the combined mechanisms of cognitive gains from role breadth self-efficacy and affective costs from emotional exhaustion, whereas the overall indirect effect on avoidance job crafting is non-significant. (4) Idiosyncratic deals (i-deals) function as a dynamic boundary mechanism that amplifies the positive impact of role breadth self-efficacy and mitigates the negative effects of emotional exhaustion, while moderating the mediating roles of both pathways. This research develops a dual-path integrated model of perceived overqualification and job crafting by classifying job crafting categories, incorporating cognitive–affective pathways, and introducing i-deals as a contextual element. These findings respond to scholarly demands for elucidating the intricate connections between perceived overqualification and job crafting through integrative perspectives; in addition, they offer theoretical and practical insights for organizations to leverage the potential of overqualified individuals appropriately.

## 1. Introduction

Recent years have witnessed intensified global economic divergence and a transformation of China’s economy, resulting in heightened competition in the labor market and underscoring structural employment contradictions and talent mismatch issues. The devaluation of degrees resulting from the extensive proliferation of higher education intensifies supply–demand disparities. By 2025, the college graduate population in China is expected to reach 12.22 million, reflecting an annual increase of 430,000 (the 2025 national conference on the employment and entrepreneurship of graduates from regular institutions of higher learning was held (Ministry of Education of the People’s Republic of China, 2025)). This trend is likely to intensify the existing “oversupply” in the labor market. In this environment, companies tend to prioritize highly qualified talent, while employees have to respond to competition through “downward-compatible” job choices or “involution-style” self-improvement, resulting in widespread talent “downskilling”. Perceived overqualification (POQ) has become an increasingly prevalent phenomenon. POQ is defined as employees’ perception that their jobs require lower knowledge and skills than they actually possess (Shang et al., 2024). Surveys revealed that approximately 50% of employees globally (and 84% in China) perceive their qualifications to surpass job requirements (M. J. Zhang et al., 2016), indicating the prevalence of perceived overqualification. Subjective perceptions of overqualification may differ from objective conditions; however, it is subjective experiences of employees that effectively predict work attitudes and behaviors (Erdogan & Bauer, 2021; Yang & Li, 2021). Therefore, perceived overqualification is an essential area for research and practice.

Current research on POQ focuses on singular perspectives, specifically negative effects represent the key scope of earlier studies that defined overqualification as a misallocation of human capital. Such misallocation cultivates perceptions of job mismatch, feelings of inequity, increased turnover intentions, and lower job satisfaction (Erdogan et al., 2018). Counterproductive behaviors such as cyberloafing, work withdrawal, and workplace deviance may also result from it (M. Wang et al., 2025; W. Zhang et al., 2024). However, positive organizational scholarship indicates that high qualifications can elicit proactive cognitive appraisals. These appraisals may then transform into self-development drivers, motivating employees towards constructive behaviors such as innovation, organizational citizenship, and bootleg innovation (Hu et al., 2015; Luksyte & Spitzmueller, 2016; Ma & Wang, 2024; Shang et al., 2024). These contradictions suggest that perceived overqualification operates as a double-edged sword. Its effects are contingent upon how employees cognitively appraise and affectively react to job-person mismatch (Yang & Li, 2021). Existing research, however, has primarily utilized single-path explanations (e.g., cognitive or affective mechanisms). Therefore, it is often insufficient in explaining the interaction of these parallel processes in driving divergent behavioral outcomes (P. Li et al., 2021). An integrative framework is therefore needed to reconcile the dual effects of perceived overqualification due to this theoretical fragmentation. A promising perspective is offered by the Cognitive–Affective Personality System (CAPS) theory. This theory hypothesizes that situational features (including both external organizational contexts and internal psychological states) simultaneously activate individuals’ cognitive and affective systems, which jointly shape behavioral responses (Walter & Yuichi, 1995). Moreover, the relative dominance of cognitive versus affective unit activation determines ultimate behavioral choices (Walter & Yuichi, 1995).

Job crafting refers to the proactive modifications made by employees to their job content and responsibilities from a bottom–up perspective (Dutton, 2001; R. Liu & Yin, 2024). This concept comprises both approach and avoidance behavioral dimensions (Bruning & Campion, 2018). The mismatch in overqualification can be mitigated by this adaptive behavior; in addition, it offers a critical perspective through which to analyze the dual-edged effects of perceived overqualification. When employees are effectively incentivized to improve job-person fit, negative outcomes may be mitigated, and their latent potential can be unlocked (Xie et al., 2015). Thus, directing POQ employees toward approach job crafting has emerged as a pivotal organizational challenge. However, achieving academic consensus regarding the relationships between POQ and job crafting continues to be elusive. It is contended by certain studies that employees with perceived overqualification often lower their job crafting efforts. This reduction aims to restore psychological balance which is disrupted by perceived breaches of psychological contracts and inequity (Z. Wang et al., 2019). Conversely, others propose that employees who feel overqualified leverage their qualifications to actively improve job–person fit through proactive job crafting (F. Zhang et al., 2021). A nonlinear mechanism has also been proposed, indicating that moderate POQ fosters job crafting via resource accumulation, while excessive POQ triggers emotional depletion and inhibits such efforts (Woo, 2020). These inconsistencies may arise from three gaps: (1) Prior studies often do not distinguish between approach job crafting (motivated by growth) and avoidance job crafting (driven by stress avoidance), despite their divergent motivational roots; (2) Integrated perspectives are frequently neglected in existing research, which primarily isolates cognitive or affective pathways (P. Li et al., 2021); thus, the fundamental question regarding whether perceived overqualification exerts overall positive or negative effects on job crafting lacks adequate resolution; (3) The role of contextual factors, such as organizational interventions, remain understudied in the dynamics between POQ and job crafting.

To address the aforementioned gaps, this study puts forward a dual-path model based on the Cognitive–Affective Personality System theory. The theory posits that situational features dynamically drive behavioral choices by activating individuals’ parallel cognitive and affective processing units (Walter & Yuichi, 1995). More specifically, perceived overqualification, understood as an internal psychological state of perceived person–job misfit, can have two primary effects: (1) it may enhance employees’ positive cognitive appraisals of their capabilities and role expansion (Chu et al., 2021; M. J. Zhang et al., 2016), which amounts to role breadth self-efficacy (RBSE, cognitive unit), and (2) it can trigger negative emotional responses such as anger, boredom, and anxiety (Arvan et al., 2022; Chen et al., 2017; Peng et al., 2023), thereby exacerbating emotional exhaustion (EE, affective unit). Approach and avoidance job crafting are differentially driven by these dual psychological mechanisms. Along the cognitive pathway, POQ elevates role breadth self-efficacy, thereby bolstering confidence to pursue approach crafting and inhibiting avoidance behaviors. Along the affective pathway, POQ intensifies emotional exhaustion, suppressing approach crafting and amplifying avoidance tendencies. The capacity of situational interventions to modulate cognitive–affective dynamics is also emphasized by the CAPS theory (Walter & Yuichi, 1995). This premise underpins our introduction of idiosyncratic deals (i-deals) as a contextual intervention mechanism; i-deals are designed to recalibrate employees’ psychological responses and behavioral choices regarding POQ. In organizations, overqualified employees often represent underutilized high-quality resources. I-deals refer to mutually beneficial, negotiated work arrangements featuring customized terms between organizations and employees (D. Rousseau, 2001). Such i-deals, when tailored for these high-potential employees, have the capability to enhance positive cognitive appraisals while mitigating negative affective reactions, ultimately fostering proactive work behaviors.

In summary, this study develops a dual-path integrated model grounded in the Cognitive–Affective Personality System theory to resolve three core questions: (1) Does POQ drive approach and avoidance job crafting? (2) What cognitive and affective mechanisms underlie these effects? (3) Under what boundary conditions do these pathways yield positive outcomes? This research seeks to offer three key contributions through addressing these questions. (1) It integrates theoretical perspectives. The introduction of CAPS theory into the study of POQ and job crafting allows for the construction of a parallel cognitive–affective mediation mechanism. This mechanism indicates the competitive interaction between cognitive and affective pathways, and such a dynamic framework helps reconcile paradoxical behavioral outcomes, thereby advancing a holistic understanding of POQ’s dual-edged nature. (2) The study achieves contextual boundary expansion. It elucidates how idiosyncratic deals (i-deals) serve as contextual interventions that differentially moderate the dual pathways, which expands the boundary conditions for how perceived overqualification influences job crafting. (3) Practical implications are offered. This research offers actionable guidance for organizations to implement targeted interventions that unlock the potential of overqualified employees.

## 2. Theoretical Foundation and Research Hypotheses

### 2.1. Theoretical Framework

The Cognitive–Affective Personality System (CAPS) theory integrates situational features (including both external contexts and internal psychological milieus), individual personality systems (cognitive and affective units), and behavioral responses into a unified theoretical framework (Walter & Yuichi, 1995), providing comprehensive and fitting theoretical support for this study. This theory hypothesizes that rational cognition and emotional impulses fulfill critical mediating roles as situational stimuli transform into behavioral responses (Walter & Yuichi, 1995). As an internal psychological context of person–job mismatch, perceived overqualification (POQ) can also trigger cognitive perceptions and affective experiences in employees (Yang & Li, 2021). Therefore, these responses may cultivate approach and avoidance behavioral strategies, respectively. Accordingly, this study positions role breadth self-efficacy (RBSE) and emotional exhaustion (EE) as mediating variables. These variables illustrate the mechanism by which POQ influences two types of job crafting behaviors. From a cognitive viewpoint, POQ cultivates positive self-perceptions concerning capability and role expansion (i.e., RBSE) (M. J. Zhang et al., 2016). This process strengthens employees’ motivation for approach crafting but also suppresses their avoidance behaviors. From an affective standpoint, employees who perceive overqualification tend to develop negative emotional experiences due to the “over-capability-under-position” dissonance (Arvan et al., 2022), which then leads to emotional exhaustion. Emotional exhaustion, accordingly, inhibits approach crafting and amplifies avoidance tendencies.

Furthermore, the CAPS theory also emphasizes that various interactive contexts in organizational environments act as external stimuli. These stimuli significantly influence individuals’ cognitive evaluations and emotional reactions (Walter & Yuichi, 1995). Under the CAPS framework, idiosyncratic deals (i-deals) as external contextual support can alter individuals’ encoding processes and psychological responses to overqualification mismatch (Ng, 2017), thereby affecting the processing patterns of cognitive and affective units and generating corresponding behavioral outcomes. This study therefore proposes i-deals as a contextual intervention mechanism. It is hypothesized that i-deals exert positive moderating effects through dual pathways. Cognitively, i-deals can align job demands with employee competencies to amplify POQ’s cognitive benefits (e.g., customized tasks enhance role breadth self-efficacy). Affectively, they reduce constraints, which helps to buffer emotional costs (e.g., flexible work arrangements alleviate emotional exhaustion).

**Figure 1:**
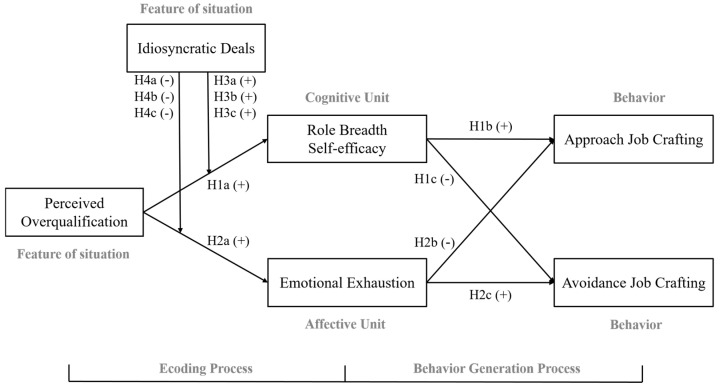
Research model.

An integrated “dual-path dual-outcome” model founded on this theoretical framework is constructed to illustrate how perceived overqualification affects job crafting. The model designates role breadth self-efficacy as the cognitive mediator and emotional exhaustion as the affective mediator while also introducing i-deals as an organizational-level moderator. Figure 1 displays our conceptual model. The annotated pathways in this model align with specific research hypotheses, which are detailed in the following sections.

### 2.2. Cognitive Pathway: The Mediating Role of Role Breadth Self-Efficacy

Perceived overqualification (POQ) arises from a mismatch between an individual’s competencies and their work environment, indicating a subjective conviction that their qualifications surpass the demands of their position (Maynard et al., 2006). According to the Cognitive–Affective Personality System (CAPS) theory, the psychological state of perceived person–job misfit activates an individual’s cognitive units (Walter & Yuichi, 1995). Employees perform a cognitive appraisal of their capabilities to fulfill innate needs for self-actualization once they acknowledge that their knowledge, skills, and experience are superior to job requirements. Role breadth self-efficacy (RBSE) denotes an individual’s confidence in their ability to expand role boundaries and undertake broader responsibilities (Z. Liu & Liu, 2024; Parker, 1998). Individuals who perceive themselves as overqualified experience a cognitive acknowledgment of their surplus capabilities, which strengthens their confidence and motivation to execute wider role-related tasks (Chu et al., 2021; M. J. Zhang et al., 2016).

Perceived overqualified employees, from a task completion perspective, typically fulfill assigned job responsibilities with relative ease (Guo et al., 2024). Proactive engagement in extra-role behaviors is often cultivated by this proficiency; examples include innovating efficiency-enhancing methods or voluntarily carrying out more complex tasks (Chu et al., 2021; Z. Wang, 2018). Such engagement corresponds with the motivational premises of RBSE. From a resource acquisition standpoint, lighter workloads and higher task proficiency reduce organizational constraints for overqualified employees (M. J. Zhang et al., 2016), granting them greater time surplus and accessible resources. This resource abundance facilitates extra-role initiatives, thereby further strengthening RBSE. Additionally, overqualified employees generally exhibit elevated self-confidence. When they successfully execute extra-role behaviors and receive organizational recognition, they tend to attribute achievements to their own abilities and efforts (Z. Wang, 2018). Such self-enhancing attributions reinforce RBSE, creating a positive self-reinforcement cycle. Based on this reasoning, we propose the following hypothesis:

**Hypothesis** **1a.** *Perceived overqualification positively influences role breadth self-efficacy.*

The concept of approach job crafting describes a proactive behavior. This behavior is represented by the active redesign of work environments, the expansion of role responsibilities, and the pursuit of resources and opportunities to better align jobs with individual capabilities and needs (Bruning & Campion, 2018). According to the CPAS theory, individuals’ cognitive evaluations shape behavioral motivations and drive corresponding actions (Walter & Yuichi, 1995). The situational feature (POQ), more specifically, activates individuals’ cognitive unit (RBSE), thereby motivating them to adopt approach or avoidance behavioral strategies. As a form of ability self-assessment, RBSE serves as a critical antecedent to behavioral change, effectively forecasting proactive behaviors and bolstering confidence in initiating such actions (M. J. Zhang et al., 2016). Employees with higher RBSE exhibit increased confidence and motivation to modify their current work conditions, thereby demonstrating greater propensity for approach job crafting. On the one hand, individuals with high RBSE possess robust confidence in their abilities and maintain an optimistic outlook toward future development (Parker, 1998). When perceiving overqualification, they are more likely to transcend predefined role boundaries through role expansion and job redesign, leveraging their full potential to bridge the gap between current tasks and ideal standards (Han, 2020), thereby mitigating perceived person–job misfit. On the other hand, job crafting inherently involves risks (e.g., resistance to role changes or resource limitations) (Fan et al., 2024). RBSE reinforces employees’ confidence in their capacity to navigate challenges and persist through setbacks (Beltrán-Martín et al., 2017; Hwang et al., 2015), providing the psychological resilience critical to sustaining approach job crafting.

Avoidance job crafting as a defensive behavioral strategy centers on mitigating workplace stress and negative demands, typically manifesting as withdrawal from specific tasks or responsibilities (Bruning & Campion, 2018). Grounded in the Cognitive–Affective Personality System theory, individuals’ positive self-perceptions motivate them to adopt strategies aligned with their cognitive schemas (Walter & Yuichi, 1995). However, avoidance job crafting, which is characterized by a passive behavioral orientation, conflicts with the proactive cognitive traits embodied by role breadth self-efficacy. Individuals with high RBSE exhibit three defining features: (1) significant confidence in addressing diverse work challenges (X. Liu et al., 2024), perceiving obstacles as manageable rather than threats requiring avoidance; (2) instead of retreating from responsibilities, they actively seek broader role engagement (Han, 2020), driven by an intrinsic belief in their multi-domain competence; (3) prioritization resource-seeking and task enrichment over defensive strategies (Strauss et al., 2009), aligning actions with their self-perceived efficacy to shape their work environment. Avoidance job crafting is, for these reasons, negatively influenced by role breadth self-efficacy.

Building on CAPS’s “feature of situation → cognitive unit → behavior” pathway, we propose that role breadth self-efficacy mediates the relationship between perceived overqualification and job crafting. Specifically, employees who perceive overqualification experience a “competence surplus” in their current positions. This surplus reinforces positive self-appraisals of their capacity to handle broader responsibilities, thereby heightening role breadth self-efficacy (Z. Liu & Liu, 2024; M. J. Zhang et al., 2016). Elevated RBSE strengthens agentic motivations to reshape work proactively (approach job crafting) while dampening defensive tendencies to withdraw (avoidance job crafting), as previously deduced. Thus, we hypothesize the following:

**Hypothesis** **1b.** *Perceived overqualification positively influences approach job crafting through the mediating role of role breadth self-efficacy.*

**Hypothesis** **1c.** *Perceived overqualification negatively influences avoidance job crafting through the mediating role of role breadth self-efficacy.*

### 2.3. Affective Pathway: The Mediating Role of Emotional Exhaustion

Emotional exhaustion, indicative of diminished psychological resources in occupational settings (Maslach & Jackson, 1981), manifests as a notable reduction in initiative and enthusiasm, coupled with persistent depletion of emotional reserves (C. Li & Shi, 2003). According to the Cognitive–Affective Personality System theory (Walter & Yuichi, 1995), perceived overqualification, as an internal psychological situation of perceived person–job misfit, may trigger affective experiences that influence subsequent behaviors. Employees with perceived overqualification, situated in a state of person–job mismatch, experience unmet needs and aspirations related to skill utilization, challenge-seeking, and achievement fulfillment in their current roles, resulting in pervasive negative emotional outcomes. Specifically, perceived overqualification induces disillusionment and boredom due to monotonous, repetitive tasks that fail to provide a sense of accomplishment (Yang & Li, 2021) while simultaneously triggering anxiety about stagnant skill development and future career prospects (Dong et al., 2023; W. Zhang et al., 2022). Furthermore, such employees may perceive their roles as waste of personal resources (e.g., knowledge, skills, time), fostering anger through perceived inequity in the effort–reward balance (Peng et al., 2023). As these negative emotions—boredom, disillusionment, anxiety, and anger—persist amid unchanging overqualification, employees increasingly invest emotional and psychological resources to manage this distress. When these resources are fully depleted, emotional exhaustion ensues. Thus, we hypothesize the following:

**Hypothesis** **2a.** *Perceived overqualification positively influences emotional exhaustion.*

Emotional exhaustion, a critical affective state within the CAPS framework (Walter & Yuichi, 1995), shapes behavioral motivation by depleting individuals’ psychological resources and emotion-driven actions. Specifically, the situational feature (POQ) activates individuals’ affective unit (emotional exhaustion), thereby motivating them to adopt approach or avoidance behavioral strategies. Employees experiencing emotional exhaustion demonstrate a decline in initiative and heightened aversion to workplace changes, as their depleted emotional reserves diminish their capacity for proactive engagement. Specifically, approach job crafting, which involves positive behavioral changes such as expanding role boundaries, applying new knowledge or technologies (Xin et al., 2024), and fostering professional networks, requires sustained energy and optimism—qualities often absent in emotionally exhausted employees. Instead, driven by a motivation to alleviate emotional strain, they tend to preserve the status quo or adopt withdrawal strategies (Yao et al., 2024). Conversely, avoidance job crafting, characterized by reducing task complexity, limiting social interactions, and simplifying responsibilities, aligns with emotionally exhausted employees’ desire to mitigate stressors and regain control over their work environment. This divergent behavioral pattern arises because emotional exhaustion amplifies defensive coping mechanisms while suppressing proactive behaviors.

Building upon CAPS’s “feature of situation → affective unit → behavior” pathway, we propose that emotional exhaustion serves as a mediator in the relationship between perceived overqualification and job crafting. Prolonged negative emotions (e.g., boredom, anxiety, resentment) stemming from perceived overqualification (a situational mismatch where employees feel “underemployed” or “underutilized”) escalate into chronic emotional exhaustion. This exhaustion affects job crafting in distinct ways: it depletes the psychological resources necessary for approach job crafting (e.g., enthusiasm for innovation), thereby suppressing such behaviors, and it intensifies avoidance job crafting as a self-protective response to evade further stress. Thus, we hypothesize the following:

**Hypothesis** **2b.** *Perceived overqualification negatively influences approach job crafting through the mediating role of emotional exhaustion.*

**Hypothesis** **2c.** *Perceived overqualification positively influences avoidance job crafting through the mediating role of emotional exhaustion.*

### 2.4. The Moderating Role of Idiosyncratic Deals in the Cognitive Pathway

I-deals, defined as voluntary, customized, and flexible employment agreements negotiated between organizations and employees to mutually fulfill both parties’ interests (D. M. Rousseau et al., 2006), encompass personalized job tasks, specialized compensation incentives, and flexible work arrangements (Rosen et al., 2013). The Cognitive–Affective Personality System (CAPS) theory posits that interactions with the organizational environment, as external stimuli, play a crucial role in shaping individuals’ cognitive appraisals and emotional responses (Walter & Yuichi, 1995). As external contextual resources provided by the organization, i-deals can resonate with the psychological internal context of overqualified employees, serving to alleviate person–job mismatches while fostering employees’ positive cognitive evaluations and emotional attachment to the organization (Ng, 2017). Therefore, i-deals are introduced in this study as a moderating variable to investigate how they shape the mechanisms that underlie the impact of perceived overqualification on job crafting.

From the cognitive pathway perspective, as a situational amplifier, i-deals negotiated between organizations and employees can enhance employees’ cognitive schemas of “my qualifications are valued and utilizable”. This reinforcement strengthens the positive effect of perceived overqualification on role breadth self-efficacy. Specifically, in terms of job tasks, i-deals enhance the autonomy, diversity, and flexibility of work content by providing tailored opportunities and assigning more challenging responsibilities (Hornung et al., 2019; Wei et al., 2010). This arrangement enables employees who feel overqualified to apply their surplus competencies, thereby strengthening role breadth self-efficacy as they extend job boundaries. Regarding time and location flexibility, i-deals empower employees with autonomy over scheduling and work environments, reducing role constraints and bolstering their sense of control and role breadth self-efficacy through improved work–life integration. In the domain of compensation design, i-deals surpass traditional rigid systems by rewarding employees for leveraging their surplus skills (e.g., expertise, knowledge) to create unique organizational value. This incentive mechanism reinforces employees’ confidence in achieving personal and professional goals through work, further motivating them to pursue broader roles and responsibilities. Based on this reasoning, we hypothesize the following:

**Hypothesis** **3a.** *Idiosyncratic deals strengthen the positive relationship between perceived overqualification and role breadth self-efficacy; specifically, the stronger the i-deals negotiated, the stronger the positive effect of perceived overqualification on role breadth self-efficacy, and vice versa.*

Further, this study proposes that i-deals moderate the mediating function of role breadth self-efficacy. Based on the Cognitive–Affective Personality System theory, the interaction between situational features and individuals’ cognitive units plays a pivotal role in shaping behavioral choices (Walter & Yuichi, 1995). When organizations provide overqualified employees with i-deals that include customized job arrangements, incentivized compensation, challenging tasks, and adaptive work conditions, these employees tend to perceive their surplus competencies as being effectively utilized. This perception enhances their confidence to undertake broader roles (i.e., higher role breadth self-efficacy), which in turn motivates proactive efforts to reshape their work and resolve mismatches. As a result, employees with increased role breadth self-efficacy are more likely to engage in approach job crafting alongside a reduction in avoidance strategies. Building on H3a, we hypothesize moderated mediation effects:

**Hypothesis** **3b.** *Idiosyncratic deals positively moderate the mediating effect of role breadth self-efficacy on the relationship between perceived overqualification and approach job crafting, such that the stronger the i-deals negotiated, the stronger the positive indirect effect of perceived overqualification on approach job crafting via role breadth self-efficacy, and vice versa.*

**Hypothesis** **3c.** *Idiosyncratic deals positively moderate the mediating effect of role breadth self-efficacy on the relationship between perceived overqualification and avoidance job crafting, such that the stronger the i-deals negotiated, the stronger the negative indirect effect of perceived overqualification on avoidance job crafting via role breadth self-efficacy, and vice versa.*

### 2.5. The Moderating Role of Idiosyncratic Deals in the Affective Pathway

Considering the affective perspective, idiosyncratic deals (i-deals) negotiated between organizations and employees function as a situational buffer. This buffer redirects employees’ focus away from unfulfilled aspirations, enabling them to reinterpret perceived overqualification through the lens of organizational support (“my needs are acknowledged”). The negative spillover consequences of perceived overqualification on emotional exhaustion are effectively lessened by this cognitive–affective recalibration. Specifically, regarding job tasks, i-deals enhance the autonomy and flexibility of work content, methods, and processes. Such improvements alleviate boredom and anxiety among overqualified employees by allowing them to redesign their roles (Hornung et al., 2014). In terms of time and location flexibility, the tailored arrangements inherent in i-deals enable employees to expand job boundaries, achieve work–family balance, and reduce feelings of relative deprivation and unfairness, thereby counteracting negative emotions triggered by perceived overqualification (Call et al., 2015). When compensation incentives in i-deals are negotiated to align with employees’ contributions, they cultivate trust; this trust ensures that effort and expertise receive fair rewards, which lessens career-related anxiety and insecurity (Liao et al., 2016). Collectively, these differentiated strategies help suppress the emergence of negative emotions, reducing the likelihood of employees spiraling into emotional exhaustion. Based on this reasoning, we hypothesize the following:

**Hypothesis** **4a.** *Idiosyncratic deals attenuate the positive relationship between perceived overqualification and emotional exhaustion; specifically, the stronger the i-deals negotiated, the weaker the positive effect of perceived overqualification on emotional exhaustion, and vice versa.*

We also propose, extending this logic, that i-deals moderate the mediating role of emotional exhaustion. Based on the Cognitive–Affective Personality System theory, the interplay between situational features and individuals’ affective units fundamentally shapes behavioral choices (Walter & Yuichi, 1995). By providing adaptive arrangements that align with personal needs and expectations, i-deals minimize the accumulation of negative emotions and lower emotional exhaustion among overqualified employees. This reduction in exhaustion enhances employee capacity to pursue approach job crafting while curbing avoidance strategies. Integrating H4a, we hypothesize moderated mediation mechanisms:

**Hypothesis** **4b.** *Idiosyncratic deals negatively moderate the mediating effect of emotional exhaustion on the relationship between perceived overqualification and approach job crafting, such that the stronger the i-deals negotiated, the weaker the negative indirect effect of perceived overqualification on approach job crafting via emotional exhaustion, and vice versa.*

**Hypothesis** **4c.** *Idiosyncratic deals negatively moderate the mediating effect of emotional exhaustion on the relationship between perceived overqualification and avoidance job crafting, such that the stronger the i-deals negotiated, the weaker the positive indirect effect of perceived overqualification on avoidance job crafting via emotional exhaustion, and vice versa.*

## 3. Materials and Methods

### 3.1. Sample and Procedures

Questionnaires were distributed through both online and offline channels, and the Wenjuanxing platform (a widely used Chinese survey tool) was utilized for all data collection. On the one hand, human resource department managers from organizations were contacted via phone calls and emails. After obtaining consent, electronic survey links were randomly distributed to employees. On the other hand, working professionals within the authors’ personal networks (e.g., friends, family members, and colleagues) were invited to participate, and the survey was further disseminated through snowball sampling. To ensure broader sample diversity and improved representativeness, initial respondents were asked to recommend potential participants with the following characteristics: (1) colleagues from different departments; (2) a difference in work experience of more than two years; and (3) different educational levels. To mitigate common method bias, data were collected in two phases. In Phase 1 (May 2024), 700 questionnaires were distributed, yielding 651 valid responses. This phase focused on measuring perceived overqualification, idiosyncratic deals, and demographic variables (e.g., age, gender). In Phase 2 (July 2024), the same 651 participants received follow-up questionnaires, achieving 597 valid responses. This phase targeted role breadth self-efficacy, emotional exhaustion, approach job crafting, and avoidance job crafting. After pairing data from the two phases and eliminating invalid responses (e.g., patterned answers, completion time < 120 s), a final sample of 556 participants was retained for analysis.

Among the 556 valid participants, 43.53% identified as male and 56.47% as female. Regarding age, 37.77% were 25 years old or younger, 42.81% were between 26 and 35 years old, and 19.42% were 36 years old or above. In terms of educational attainment, 86.33% held a bachelor’s degree or higher, including master’s and doctoral degrees. For marital status, 63.67% were unmarried, while 36.33% were married. With respect to work experience, 61.15% had three years or less of tenure, and 38.85% had more than three years. In the distribution of job categories, 38.85% worked in administrative/functional roles, 23.74% in professional/technical roles, 18.18% in marketing/sales roles, 10.97% in production/operations roles, and 8.27% in other roles. Hierarchically, 56.29% were non-managerial employees, 24.28% were frontline supervisors, and 19.42% were middle-to-senior managers. Participants were employed across diverse organizational types: 24.82% in state-owned enterprises, 41.19% in private enterprises, 10.79% in joint-venture/foreign-funded companies, 13.85% in government/public institutions, and 9.35% in other sectors. Overall, the sample exhibited balanced diversity across demographic categories, hierarchical levels, and organizational contexts. The high proportion of highly educated individuals aligns with the study’s focus, as prior research suggests that those with advanced education are more prone to perceive overqualification in situations of person–job mismatch. This characteristic underscores the validity of the sample for addressing the research objectives.

### 3.2. Measurement

Well-validated scales, widely recognized in prior research and once applied in the Chinese context, were employed in this study. To enhance contextual applicability in Chinese organizational settings for Western-developed scales originally situated in non-Chinese contexts, translation and contextual adaptation of measurement items were conducted; this was achieved through referencing domestic localization studies and adhering to Chinese linguistic conventions. The questionnaire was designed using a five-point Likert scale, ranging from 1 (“Strongly Disagree”) to 5 (“Strongly Agree”), with higher scores indicating greater agreement. The questionnaire is presented in Appendix A.

Perceived Overqualification was measured with the unidimensional 9-item scale developed by Maynard et al. (2006). A sample item includes “My skill level exceeds what is required to perform my job”.

Role Breadth Self-Efficacy was assessed using Parker et al.’s (2006) 7-item unidimensional scale. A representative item is “I can design new procedures for my work area”. 

Emotional Exhaustion was evaluated through a unidimensional 5-item scale translated and adapted by Chinese scholars C. Li and Shi (2003). A sample item states “My job leaves me feeling emotionally and physically drained”.

Approach Job Crafting was measured using Bruning and Campion’s (2018) 23-item scale comprising five dimensions: work organization, adaptability, metacognition, role expansion, and social expansion. An example item is “I apply new knowledge or technology to automate tasks and enhance efficiency”.

Avoidance Job Crafting was captured via Bruning and Campion’s (2018) 7-item scale, including dimensions of role reduction and withdrawal. A sample item reads “I find ways to avoid time-consuming work tasks”.

Idiosyncratic Deals were assessed using Rosen et al.’s (2013) 16-item scale with four dimensions: task customization, schedule flexibility, location flexibility, and compensation incentives. An example item is “My supervisor considers my personal needs when assigning work schedules”.

Prior studies suggest that demographic differences (e.g., gender, age, education) may influence perceived overqualification and job crafting behaviors. To isolate the effects of core variables, this study controlled for employees’ gender, age, education, marital status, years of work, position category, position level, and nature of organization in the analysis. These controls ensure that observed relationships are not confounded by extraneous demographic factors.

## 4. Analysis and Results

### 4.1. Reliability and Validity

This study employed SPSS 26.0 and AMOS 24.0 for reliability and validity tests. The analysis revealed Cronbach’s α coefficients of 0.905 for perceived overqualification, 0.901 for role breadth self-efficacy, 0.914 for emotional exhaustion, 0.974 for approach job crafting, 0.902 for avoidance job crafting, and 0.950 for idiosyncratic deals, all exceeding the 0.9 threshold, indicating good reliability of the scales. Confirmatory factor analysis (CFA) fit indices for each construct are presented in Table 1. Satisfactory model fit was demonstrated by all scales, with the exception of avoidance job crafting. For avoidance job crafting, the fit indices were χ^2^/df = 4.744, RMSEA = 0.082, TLI = 0.964, CFI = 0.983, and IFI = 0.983. According to the evaluation criteria proposed by Qiu and Lin (2009) (where χ^2^/df < 5 and RMSEA < 0.1 are acceptable), the model fit for avoidance job crafting remains in an acceptable range. In addition, Table 2 shows that the second-order six-factor model achieved optimal fit indices (χ^2^/df = 1.646, RMSEA = 0.034, TLI = 0.947, CFI = 0.950, IFI = 0.950), confirming the six constructs as independent factors with satisfactory construct validity.

Convergent validity was assessed through standardized factor loadings, average variance extracted (AVE), and composite reliability (CR). All standardized factor loadings for the items were above 0.5. The AVE values were as follows: 0.515 for perceived overqualification, 0.567 for role breadth self-efficacy, 0.683 for emotional exhaustion, 0.890 for approach job crafting, 0.828 for avoidance job crafting, and 0.842 for idiosyncratic deals. The corresponding CR values were 0.905, 0.901, 0.915, 0.976, 0.906, and 0.955, respectively. With all AVE values above 0.5 and CR values exceeding 0.9, the scales demonstrated adequate convergent validity. Furthermore, discriminant validity was established as the absolute values of correlation coefficients among variables were lower than the square roots of their respective AVE values (Table 3).

### 4.2. Common Method Variance

This study employed Harman’s single-factor test for principal component analysis, extracting a total of seven factors that accounted for 64.515% of the total variance. The first factor explained a maximum variance of 30.998%, which is less than 40%, providing preliminary evidence that there is no serious common method bias. Furthermore, this study conducted an unmeasured latent method factor test by adding a common method factor to compare the model fit with the original model. The results showed that the inclusion of the common method factor did not significantly improve the model fit (χ^2^/df = 1.617, RMSEA = 0.033, TLI = 0.950, CFI = 0.953, IFI = 0.953), further indicating that there is no serious common method bias in this study.

### 4.3. Correlation Analysis

SPSS 26.0 software was utilized in this study to analyze the correlations among perceived overqualification, role breadth self-efficacy, emotional exhaustion, approach job crafting, avoidance job crafting, and idiosyncratic deals. Table 3 presents the means, standard deviations, and correlation coefficients for each variable. Perceived overqualification was found to be significantly positively correlated with role breadth self-efficacy (r = 0.335, *p* < 0.01), providing preliminary support for hypothesis H1a. Additionally, perceived overqualification showed a significant positive correlation with emotional exhaustion (r = 0.132, *p* < 0.01), offering initial evidence for hypothesis H2a. These results partially validate some of the hypotheses and lay the groundwork for subsequent analyses.

### 4.4. Hypothesis Testing

#### 4.4.1. Mediating Effects Analysis

This study constructed a structural equation model comprising five variables to investigate four mediating pathways. Structural equation modeling (SEM) was chosen because it is particularly effective in clarifying causal relationships between factors, aligning with the recommendations of Wen and Ye (2014). Parceling was conducted by this study for the five dimensions of approach job crafting and the two dimensions of avoidance job crafting. This procedure was adopted as both are second-order latent variables, following the recommendations of Wu and Wen (2011).

**Figure 2:**
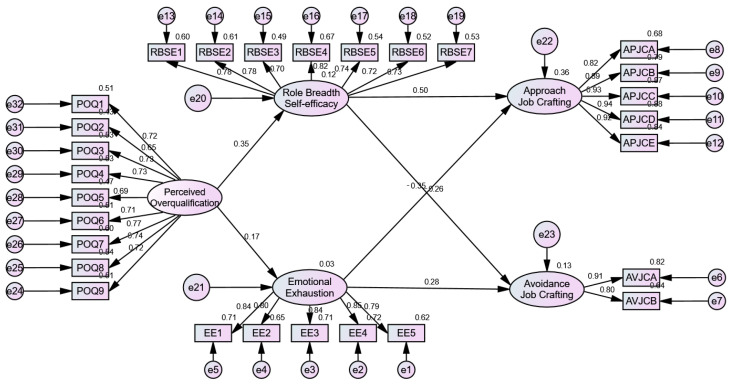
Path fitting of the hypothesis model (standardized coefficient).

The results revealed that the fit indices of the structural equation model were χ^2^/df = 2.913, RMSEA = 0.059, TLI = 0.931, CFI = 0.937, and IFI = 0.937, indicating an acceptable model fit. The path coefficients presented in Table 4 and Figure 2 demonstrate that perceived overqualification has a significant positive impact on role breadth self-efficacy (β = 0.345, *p* < 0.001), thereby validating hypothesis H1a. Additionally, perceived overqualification significantly positively affects emotional exhaustion (β = 0.171, *p* < 0.001), thus confirming hypothesis H2a.

Subsequently, for the analysis of mediation effects, the random sampling was set by this study to 5000 iterations, and the Bootstrap mediation test procedure in AMOS 24.0 was utilized, with the results presented in Table 5. For approach job crafting, role breadth self-efficacy (RBSE) significantly mediated the relationship between perceived overqualification (POQ) and approach job crafting [β = 0.174, CI (0.129, 0.228), excluding zero], thereby supporting Hypothesis H1b. Emotional exhaustion (EE) also exerted a significant mediating effect in the relationship between POQ and approach job crafting [β = −0.060, CI (−0.111, −0.021), excluding zero], confirming Hypothesis H2b. The total indirect effect of POQ on approach job crafting through RBSE and EE was statistically significant [β = 0.085, CI (0.053, 0.120), excluding zero]. These results demonstrate that RBSE and EE act as dual mediators between POQ and approach job crafting, with the overall indirect effect being positive. This indicates that the cognitive pathway (via RBSE) dominates over the affective pathway (via EE) in shaping approach behaviors.

For avoidance job crafting, role breadth self-efficacy significantly mediated the relationship between perceived overqualification and avoidance job crafting [β = −0.089, CI (−0.133, −0.055), excluding zero], supporting Hypothesis H1c. Emotional exhaustion also showed a significant mediating effect [β = 0.047, CI (0.018, 0.091), excluding zero], validating Hypothesis H2c. However, the total indirect effect of POQ on avoidance job crafting through RBSE and EE was non-significant [β = −0.013, CI (−0.038, 0.001), including zero]. This suggests that while both RBSE and EE independently mediate the relationship between POQ and avoidance job crafting, their opposing directional effects (RBSE suppressing avoidance, EE promoting avoidance) cancel each other out at the aggregate level, resulting in a net non-significant indirect effect.

#### 4.4.2. Moderating Effects Analysis

To test the moderating effects, the hierarchical regression method, proposed by Baron and Kenny (X. Li et al., 2021), was employed in this study. To examine the moderating role of idiosyncratic deals in the relationship between perceived overqualification and role breadth self-efficacy, as proposed in H3a, Models 1–3 were constructed for validation. Similarly, to investigate the moderating role of idiosyncratic deals in the relationship between perceived overqualification and emotional exhaustion, as proposed in H4a, Models 4–6 were developed for validation, as shown in Table 6.

The results indicate that the interaction term between perceived overqualification and idiosyncratic deals has a significant impact on role breadth self-efficacy (β = 0.092, *p* < 0.05), suggesting that idiosyncratic deals play a significant moderating role in the relationship between perceived overqualification and role breadth self-efficacy, thereby supporting H3a. Additionally, the interaction term between perceived overqualification and idiosyncratic deals significantly affects emotional exhaustion (β = −0.101, *p* < 0.05), indicating that idiosyncratic deals also significantly moderate the relationship between perceived overqualification and emotional exhaustion, thus supporting H4a.

**Figure 3:**
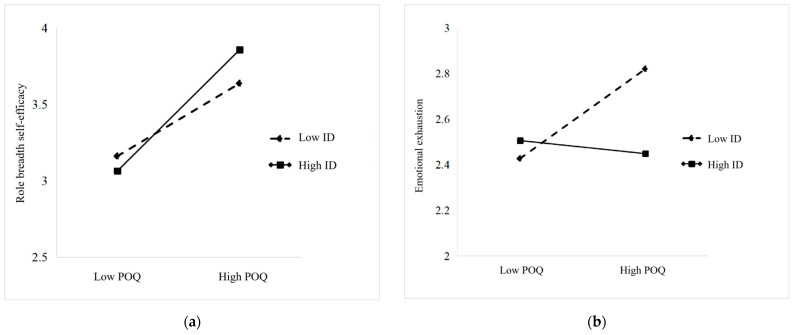
Moderating effect. (**a**) Moderating effect of i-deals on relationship between POQ and RBSE. (**b**) Moderating effect of i-deals on relationship between POQ and EE.

To more intuitively illustrate the moderating effects of idiosyncratic deals, this study divided the sample into high and low idiosyncratic deals groups by adding and subtracting one standard deviation from the mean of idiosyncratic deals. Simple slope graphs were then plotted to depict the effects of perceived overqualification on role breadth self-efficacy and emotional exhaustion under high and low levels of idiosyncratic deals, as shown in Figure 3.

As illustrated in Figure 3a, under high levels of idiosyncratic deals, role breadth self-efficacy increases with the rise in perceived overqualification (β = 0.378, *p* < 0.001). Conversely, under low levels of idiosyncratic deals, although role breadth self-efficacy still increases with perceived overqualification (β = 0.256, *p* < 0.001), the effect size decreases from 0.378 to 0.256. This result indicates that idiosyncratic deals can enhance the positive impact of perceived overqualification on role breadth self-efficacy, further validating H3a. As depicted in Figure 3b, under low levels of idiosyncratic deals, emotional exhaustion increases with the rise in perceived overqualification (β = 0.171, *p* < 0.05). However, under high levels of idiosyncratic deals, the effect of perceived overqualification on emotional exhaustion is not significant (β = −0.003, *p* > 0.05). This result suggests that idiosyncratic deals can mitigate the positive impact of perceived overqualification on emotional exhaustion, further supporting H4a.

#### 4.4.3. Moderated Mediation Effects Analysis

The PROCESS macro program was utilized in this study, and the Bootstrap method was employed to test the moderated mediation effects, with the results presented in Table 7. As shown in Table 7, when idiosyncratic deals were one standard deviation below the mean, role breadth self-efficacy significantly mediated the relationship between perceived overqualification and approach job crafting [β = 0.158, CI (0.092, 0.223), excluding zero]. When idiosyncratic deals were one standard deviation above the mean, role breadth self-efficacy also significantly mediated this relationship [β = 0.234, CI (0.172, 0.297), excluding zero]. These findings indicate that the indirect effect of perceived overqualification on approach job crafting through role breadth self-efficacy is moderated by idiosyncratic deals, thereby supporting H3b. Similarly, the indirect effect of perceived overqualification on avoidance job crafting through role breadth self-efficacy is also moderated by idiosyncratic deals, supporting H3c.

When idiosyncratic deals were one standard deviation below the mean, emotional exhaustion significantly mediated the relationship between perceived overqualification and approach-oriented job crafting [β = −0.032, CI (−0.064, −0.005), excluding zero]. However, when idiosyncratic deals were one standard deviation above the mean, this indirect effect was not significant [β = 0.001, CI (−0.019, 0.026), including zero]. Since the effect is significant at low levels but not at high levels, the moderated mediation index was used for further evaluation. The results showed that the moderated mediation index = 0.021, 95% confidence interval [0.001, 0.048], excluding zero. This suggests that the difference in indirect effects is significant at different levels of idiosyncratic deals. Thus, the indirect effect of perceived overqualification on approach job crafting through emotional exhaustion is moderated by idiosyncratic deals, thereby supporting H4b.

When idiosyncratic deals were one standard deviation below the mean, emotional exhaustion significantly mediated the relationship between perceived overqualification and avoidance job crafting [β= 0.030, CI (0.005, 0.061), excluding zero]. However, when idiosyncratic deals were one standard deviation above the mean, this indirect effect was not significant [β = −0.001, CI (−0.025, 0.019), including zero]. Further, the moderated mediation index = −0.020, 95% confidence interval [−0.046, −0.019], excluding zero. This indicates that the indirect effect of perceived overqualification on avoidance job crafting through emotional exhaustion is moderated by idiosyncratic deals, thereby supporting H4c.

## 5. Discussion

This study aimed to reconcile inconsistent findings regarding the relationship between perceived overqualification and job crafting; for this purpose, an integrated “dual-pathway dual-outcome” model based on the Cognitive–Affective Personality System (CAPS) theory was constructed. Validation for all 12 proposed hypotheses was offered by the empirical results, and the principal findings are outlined as follows:

(1) Perceived overqualification differentially influences approach and avoidance job crafting through the dual pathways of “cognitive drive” and “affective inhibition”.

Perceived overqualification (POQ), as an internal psychological state of person–job mismatch, can simultaneously activate employees’ cognitive perceptions and affective experiences (Yang & Li, 2021), leading to divergent behavioral choices. Along the cognitive pathway, perceived overqualification positively promotes approach job crafting and negatively inhibits avoidance job crafting by enhancing role breadth self-efficacy (RBSE). Employees who perceive themselves as overqualified develop positive self-perceptions of capability surplus (manifested as RBSE), motivating proactive role expansion to resolve mismatch, aligning with the findings of M. J. Zhang et al. (2016). Notably, this study extends prior research by revealing RBSE’s negative impact on avoidance job crafting, which is a novel contribution to understanding inhibitory antecedents of passive work behaviors. Along the affective pathway, perceived overqualification negatively inhibits approach job crafting and positively influences avoidance job crafting by exacerbating emotional exhaustion. Overqualified employees who fixate on external job–person mismatch rather than internal self-regulation are susceptible to negative emotions, including boredom, anxiety, and anger, which can result in emotional exhaustion. This is consistent with the findings of Erdogan et al. (2018) and X. Li et al. (2021). Proactive approach behaviors are excluded by emotionally depleted employees due to resource depletion; these employees instead adopt avoidance strategies such as task simplification and social withdrawal to alleviate stress. While the positive effect of emotional exhaustion on negative behaviors such as work withdrawal has been the predominant focus of prior research (Xin et al., 2024), its influence on proactive behaviors such as approach job crafting has received less attention. This study therefore not only validates the positive impact of emotional exhaustion on avoidance job crafting but also confirms its significant negative impact on approach job crafting, addressing the call by Zeijen et al. (2018) to explore diverse antecedents of job crafting.

Building upon these findings, the interactive effects between cognitive and affective pathways were further analyzed in this study. A positive and significant overall indirect effect of perceived overqualification on approach job crafting is indicated by the results; this effect operates through the combined cognitive gain pathway of role breadth self-efficacy and the affective depletion pathway of emotional exhaustion, indicating the dominance of cognitive unit’s enhancement effects in behavioral choices. However, through the inhibitory pathway of role breadth self-efficacy and the reinforcing pathway of emotional exhaustion, perceived overqualification generates opposing effects that cancel each other out, resulting in non-significant overall indirect effects on avoidance job crafting. The competitive interaction mechanism between cognitive and affective units in CAPS theory (Walter & Yuichi, 1995) is verified by this result. To explain the non-significant effects on avoidance job crafting, dual theoretical explanations are proposed. First, behavioral inhibition may be created by the conflicting coexistence of empowerment and strain. Role breadth self-efficacy from competence surplus and emotional exhaustion from needs–abilities imbalance are also developed by overqualified employees. Psychological conflict during avoidance decision-making is triggered by this empowerment strain paradox—cognitive control suppresses withdrawal tendencies while affective fatigue reinforces avoidance motives—which amounts to statistical neutrality in behavioral outcomes. Second, floor effects due to organizational thresholds may constrain avoidance behaviors. Minimum job performance standards are mandated by workplace norms, meaning that a reduction in task engagement below functional baselines by even emotionally exhausted employees is unlikely. The oversimplified “either positive or negative” dichotomy regarding perceived overqualification is challenged by these findings; they confirm that its impact direction and intensity on job crafting depend on specific behavioral types (approach vs. avoidance) and the relative dominance of cognitive–affective pathways. The complexity of dual-path mechanisms is indicated by this research, which also demonstrates that integrating cognitive and affective pathways is necessary to interpret complex behavioral outcomes. Dynamic effects of situational features might be obscured by relying solely on single mechanisms, whereas the CAPS framework offers a theoretical tool for unraveling such complexities.

(2) Idiosyncratic deals (i-deals) serve as a positive moderator in the mechanisms of perceived overqualification.

Organizational contextual interventions can reshape individuals’ cognitive and affective responses, as hypothesized by the Cognitive–Affective Personality System theory (Walter & Yuichi, 1995). As a differentiated and flexible organizational support mechanism, i-deals significantly moderate the interaction between perceived overqualification and employees’ cognitive–affective responses. Although overqualified employees operate in a psychological context of perceived mismatch, organizations can amplify cognitive gains (enhancing role breadth self-efficacy) and buffer affective depletion (mitigating emotional exhaustion) through personalized job arrangements that provide resource-rich external contexts. This subsequently motivates overqualified employees to adopt approach job crafting for proactive breakthroughs while suppressing avoidance job crafting. This aligns with the “if–then” pattern proposed by CAPS theory (Walter & Yuichi, 1995): if contextualized support (i-deals) is provided, then the cognitive–affective system generates adaptive behaviors.

In the Chinese context, the effectiveness of i-deals can be interpreted through the cultural sensitivity of CAPS. The theory emphasizes that situational characteristics are interpreted through culturally shaped cognitive–affective filters (Walter & Yuichi, 1995). In Confucian-influenced societies valuing “people-oriented philosophy” and “harmony in diversity”, employees may perceive i-deals not merely as transactional arrangements but as symbolic gestures respecting individual uniqueness. This cultural lens amplifies i-deals’ capacity to activate positive cognitive schemas (e.g., “I am recognized”) and inhibit negative affective responses (e.g., “I feel constrained”), thereby strengthening their moderating effects within the CAPS framework. Therefore, by incorporating i-deals as a moderating variable, this study not only responds to Yang and Li ’s (2021) appeal to examine the interaction between organizational contextual factors and perceived overqualification but also demonstrates the application of ancient Chinese philosophies in contemporary human resource management practices.

### 5.1. Theoretical Implications

Theoretical understanding is advanced by this study utilizing a dual-path integrated model that elucidates the intricate relationship between perceived overqualification and job crafting; two primary theoretical contributions are offered:

(1) Integration of dual pathways within the CAPS framework

Theoretically, single-perspective limitations are transcended by this research through the introduction of the Cognitive–Affective Personality System (CAPS) theory for integrating cognitive and affective pathways. A dynamic explanatory framework for conflicting behavioral outcomes is offered by its revelation of the competitive relationship between these pathways. Previous research primarily carried out analyses of the effect of perceived overqualification on job crafting through isolated cognitive or affective mechanisms, thereby neglecting their concurrent effects (P. Li et al., 2021). Grounded in the CAPS theory, this study delineates the dynamic process whereby individuals translate perceived overqualification into distinct job crafting strategies; this occurs through dual mechanisms of cognitive appraisal and emotional regulation. Cognitive and affective pathways operate in competition, as demonstrated by the findings, and approach/avoidance job crafting are not mutually exclusive but coexist as products of cognitive–affective tension, which is a novel insight unattainable through single-path models. Therefore, the proposed “dual-pathway dual-outcome” model provides a holistic theoretical explanation for understanding the nuanced relationship between perceived overqualification and job crafting behaviors. Furthermore, the analysis of overall indirect effects uncovers the competitive dynamics between cognitive and affective pathways, resonating with CAPS’ emphasis on the dynamic interaction of cognition and emotion.

(2) Contextual moderating mechanisms within the CAPS framework

Regarding contextual mechanisms, idiosyncratic deals (i-deals) are introduced by this study as situational amplifiers/buffers within the CAPS framework; their differential moderating effects are explored to expand the boundary conditions of how perceived overqualification influences job crafting. While exploration of i-deals as moderators of work behaviors has occurred in existing research (Xiang et al., 2023), their differential impacts across cognitive and affective pathways remained unclear. This work clarifies how i-deals shape boundary conditions for approach and avoidance job crafting along both pathways, demonstrating how tailored organizational interventions tilt the cognitive–affective equilibrium toward desired outcomes. These findings not only broaden the theoretical scope of perceived overqualification research but also offer targeted theoretical guidance for organizations to optimize employee management strategies and effectively motivate overqualified employees through contextual interventions.

### 5.2. Managerial Implications

Perceived overqualification is a prevalent issue with challenges, necessitating proactive organizational responses through systematic human resource management practices to address its dual effects. Actionable insights are offered by this study, enabling organizations to leverage the positive outcomes of perceived overqualification while working to lessen its negative effects.

First, an acknowledgement of perceived overqualification’s dual nature is crucial for organizations, which should also adopt prudent matching strategies during recruitment. If person–job fit is prioritized, organizations should conduct rigorous job analyses and competency assessments to ensure alignment between candidates and job requirements, thereby minimizing perceived overqualification at its origin. If hiring highly qualified and promising employees is necessary for organizational development, organizations should engage in thorough communication with these employees, establish clear career development plans, and provide diversified support for career growth (e.g., cross-departmental rotations, internal promotions) to help them envision future growth opportunities.

Second, the implementation of training programs by organizations can enhance employees’ positive cognitive appraisals of perceived overqualification and strengthen their self-efficacy. For instance, case studies and scenario-based workshops are effective tools for demonstrating how surplus qualifications can be utilized extend beyond formal job descriptions. Employees can be guided to apply their excess skills in cross-functional collaborations, innovation initiatives, or internal consulting roles. Such interventions not only reframe perceived overqualification as an organizational asset but also stimulate proactive job crafting behaviors.

Third, proactive monitoring and addressing of emotional exhaustion, which can be triggered by perceived overqualification, are essential organizational responsibilities. The institutionalization of regular mental health assessments and targeted counseling services can aid in identifying and mitigating negative emotions. Open communication channels, such as supervisor–employee dialogues, peer support groups, or anonymous feedback platforms, can help employees voice their concerns and reduce feelings of stagnation. Cultivating an empathetic work environment allows organizations to redirect employees’ frustration into constructive work adaptation efforts, thereby achieving mutually beneficial outcomes.

Finally, idiosyncratic deals (i-deals) should be strategically designed and dynamically adjusted to maximize their moderating effects. Periodic interviews or surveys can be conducted by organizations to capture employees’ evolving preferences for work content, career development, and work–life balance. Developing tailored i-deals based on individual needs is crucial for employees who value autonomy and challenge, offering high-responsibility projects and flexible task ownership can enhance engagement, and for those prioritizing work–life balance, in which case remote work options or adjustable schedules can mitigate perceptions of wasted potential. It is essential to periodically review and refine i-deals to maintain alignment with employee aspirations and organizational objectives, thus ensuring their continued motivational effectiveness.

### 5.3. Limitations and Future Research

Notwithstanding its contributions, this study is not without certain limitations for future improvement.

Initially, the first set of limitations related to methodological constraints, as all variables were measured with self-reported assessments, which carries the potential for common method bias. Although multi-wave data collection through blended online and offline channels was implemented and statistical tests indicated no severe bias, future research should incorporate more objective evaluation methods; for instance, supervisor or peer assessments could be utilized for employees’ job crafting behaviors. Another methodological concern is that, notwithstanding the two-wave data collection, mediators (role breadth self-efficacy and emotional exhaustion) and outcomes (approach and avoidance job crafting) were measured simultaneously. Even though mediators were placed before outcome variables in the questionnaire, this design cannot fully establish temporal precedence. Therefore, our recommendation for future studies is the adoption of three-phase longitudinal designs. Finally, longitudinal and tracking studies could be combined, supplemented by case analysis, experimental methods, and meta-analysis, to cross-validate findings and ensure the robustness of conclusions.

Secondly, there are content limitations. For instance, Maltarich et al. (2011) proposed the notion of “voluntary mismatch”, positing that certain underemployed individuals might deliberately choose simpler jobs or lower positions to engage in activities of genuine interest or to allocate more time for family. Therefore, future research could benefit from exploring different types of POQ. Moreover, while this model focuses on organizational contextual moderators (i-deals) due to the relative stability of individual traits, personal characteristics such as mindfulness and proactive personality could potentially interact with situational factors to shape job crafting responses. Accordingly, the effect analysis of trait–situation interactions on the mechanisms between perceived overqualification and job crafting behaviors is a valuable area for further studies. Finally, employees predisposed to proactive job crafting may be more likely to initiate negotiations for i-deals, suggesting a potential reverse causality between the two constructs. Longitudinal designs or experimental approaches are recommended for future studies to further examine the temporal dynamics of this relationship.

Thirdly, the cultural applicability of the findings presents a limitation due to the scope of this study on variable relationships being confined to China’s Confucian culture, potentially restricting the cross-cultural generalizability of the results. Future research could test the reliability and universality of our conclusions by examining the relationship between perceived overqualification and job crafting across diverse cultural settings, using samples from different cultural backgrounds.

## Figures and Tables

**Table 1:** The CFA fit indices of each construct.

Variable	χ^2^/df	RMSEA	TLI	CFI	IFI
Perceived overqualification	2.168	0.046	0.984	0.991	0.991
Approach job crafting	1.946	0.041	0.979	0.981	0.981
Avoidance job crafting	4.744	0.082	0.964	0.983	0.983
Role breadth self-efficacy	2.496	0.052	0.985	0.994	0.994
Emotional exhaustion	2.231	0.047	0.993	0.997	0.997
Idiosyncratic deals	2.672	0.055	0.967	0.973	0.973

N = 556.

**Table 2:** The comparison results of six-factor model and competition model fitting.

Models	χ^2^/df	RMSEA	TLI	CFI	IFI
Six-factor model	1.646	0.034	0.947	0.950	0.950
POQ, RBSE, EE, APJC, AVJC, ID					
Five-factor model	1.783	0.038	0.936	0.939	0.939
POQ, RBSE, EE, APJC + AVJC, ID					
Four-factor model	2.666	0.055	0.864	0.869	0.870
POQ, RBSE + EE, APJC + AVJC, ID					
Three-factor model	3.168	0.063	0.823	0.830	0.830
POQ, RBSE + EE, APJC + AVJC + ID					
Two-factor model	3.992	0.073	0.756	0.765	0.766
POQ + RBSE + EE, APJC + AVJC + ID					
One-factor model	4.958	0.084	0.678	0.689	0.690
POQ + RBSE + EE + APJC + AVJC + ID					

N = 556; POQ, perceived overqualification; RBSE, role breadth self-efficacy; EE, emotional exhaustion; APJC, approach job crafting; AVJC, avoidance job crafting; ID, idiosyncratic deals.

**Table 3:** Means, standard deviations, and correlations between the study variables.

Variable	M	SD	1	2	3	4	5	6
1. Perceived overqualification	3.177	0.881	(0.718)					
2. Approach job crafting	3.592	0.878	−0.188 **	(0.943)				
3. Avoidance job crafting	2.766	0.880	0.152 **	−0.457 **	(0.910)			
4. Role breadth self-efficacy	3.461	0.757	0.335 **	0.440 **	−0.170 **	(0.753)		
5. Emotional exhaustion	3.038	0.983	0.132 **	−0.273 **	0.210 **	0.112 **	(0.826)	
6. Idiosyncratic deals	3.423	0.769	−0.318 **	0.502 **	−0.284 **	−0.012	−0.145 **	(0.918)

N = 556; ** *p* < 0.01; The diagonal line marked with “()” represents the arithmetic square root of the average variance extracted value AVE.

**Table 4:** The path coefficient of the hypothesis model.

Path	Unstandardized Coefficient	Standardized Coefficient	S.E.	C.R.	*p*
POQ→RBSE	0.316	0.345	0.044	7.182	***
RBSE→APJC	0.582	0.504	0.051	11.324	***
RBSE→AVJC	−0.330	−0.259	0.063	−5.276	***
POQ→EE	0.197	0.171	0.054	3.648	***
EE→APJC	−0.324	−0.353	0.038	−8.607	***
EE→AVJC	0.279	0.275	0.047	5.918	***

*** *p* < 0.001; POQ, perceived overqualification; RBSE, role breadth self-efficacy; EE, emotional exhaustion; APJC, approach job crafting; AVJC, avoidance job crafting.

**Table 5:** Standardized mediation tests.

Path	Effect Value	SE	Bias-Corrected 95%			Percenntile 95%		
			Lower	Upper	*p*	Lower	Upper	*p*
POQ→RBSE→APJC	0.174	0.025	0.129	0.228	0.000	0.127	0.224	0.000
POQ→EE→APJC	−0.060	0.023	−0.111	−0.021	0.001	−0.110	−0.021	0.002
POQ→RBSE and EE→APJC	0.085	0.017	0.053	0.122	0.000	0.053	0.120	0.000
POQ→RBSE→AVJC	−0.089	0.020	−0.133	−0.055	0.000	−0.130	−0.053	0.000
POQ→EE→AVJC	0.047	0.018	0.018	0.091	0.001	0.015	0.088	0.002
POQ→RBSE&EE→AVJC	−0.013	0.010	−0.038	0.001	0.079	−0.037	0.002	0.093

POQ, perceived overqualification; RBSE, role breadth self-efficacy; EE, emotional exhaustion; APJC, approach job crafting; AVJC, avoidance job crafting.

**Table 6:** Test results of the modulating effect of idiosyncratic deals.

Variable	Role Breadth Self-Efficacy			Emotional Exhaustion		
	Model 1	Model 2	Model 3	Model 4	Model 5	Model 6
Gender	−0.096 *	−0.080	−0.092 *	−0.021	−0.018	−0.006
Age	−0.135 *	−0.163 ***	−0.150 **	0.145 *	0.131 *	0.116 *
Educational	0.145 ***	0.154 ***	0.142 ***	0.004	0.014	0.028
Marital status	−0.006	0.039	0.030	−0.154 **	−0.136 *	−0.126 *
Years of work	0.019	0.058	0.051	0.012	0.021	0.029
Position category	−0.057	−0.063	−0.056	0.081	0.077	0.069
Position level	0.179 ***	0.125 **	0.137 **	0.171 ***	0.158 **	0.144 **
Nature of organization	−0.092 *	−0.128 ***	−0.118 **	0.066	0.046	0.035
POQ		0.382 ***	0.369 ***		0.061	0.075
ID		0.063	0.031		−0.092 *	−0.057
POQ×ID			0.092 *			−0.101 *
R^2^	0.074	0.202	0.208	0.077	0.091	0.099
ΔR^2^	0.074	0.128	0.007	0.077	0.015	0.008
F	5.427 ***	13.785 ***	13.023 ***	5.679 ***	5.480 ***	5.448 ***
ΔF	5.427 ***	43.817 ***	4.515 *	5.679 ***	4.402 *	4.744 *

* *p* < 0.05; ** *p* < 0.01; *** *p* <0.001; POQ, perceived overqualification; ID, idiosyncratic deals.

**Table 7:** The moderating mediating effect test of idiosyncratic deals.

Mediation Path	Level of Idiosyncratic Deals	Moderated Indirect Effect	Boot SE	LLCI	ULCI
POQ→RBSE→APJC	Low (M − 1SD)	0.158	0.033	0.092	0.223
	Medium (M)	0.196	0.028	0.139	0.251
	High (M + 1SD)	0.234	0.032	0.172	0.297
POQ→RBSE→AVJC	Low (M − 1SD)	−0.072	0.019	−0.113	−0.037
	Medium (M)	−0.089	0.020	−0.131	−0.053
	High (M + 1SD)	−0.107	0.023	−0.155	−0.064
POQ→EE→APJC	Low (M − 1SD)	−0.032	0.015	−0.064	−0.005
	Medium (M)	−0.016	0.009	−0.036	0.002
	High (M + 1SD)	0.001	0.011	−0.019	0.026
POQ→EE→AVJC	Low (M − 1SD)	0.030	0.014	0.005	0.061
	Medium (M)	0.015	0.009	−0.003	0.035
	High (M + 1SD)	−0.001	0.011	−0.025	0.019

POQ, perceived overqualification; RBSE, role breadth self-efficacy; EE, emotional exhaustion; APJC, approach job crafting; AVJC, avoidance job crafting.

## Data Availability

The raw data supporting the conclusions of this article will be made available by the corresponding author upon reasonable request.

## Appendix A

The following are the measurement items adopted.

**Perceived overqualification scale**

**Items**	**Questionnaire Items**
**Perceived overqualification**	My current educational level is higher than what is required for my job.
	My previous work experience has little to do with my ability to perform this job.
	Some of my work skills are not applicable to my current job position.
	People with a lower educational level than me can also handle my current job.
	The training I’ve received is not very helpful for me to perform my current job.
	Much of the knowledge I’ve acquired is not useful in my current job.
	My educational level is higher than the requirements of my job.
	Some people with less work experience than me can also do my current job.
	My ability level is higher than what is required to complete the job.

**Role breadth self-efficacy scale**

**Items**	**Questionnaire Items**
**Role breadth** **self-efficacy**	I can analyze the long-term problems faced by the company and propose solutions.
	When having meetings with senior/higher-level managers, I can effectively represent my department/team.
	I can design new work procedures in my own work area.
	I can help the organization/department/team set work goals.
	I can communicate and discuss issues with people outside the company, such as customers.
	I can speak in front of the members of the organization/department/team.
	I can visit other departments and put forward suggestions for their reference.

**Emotional exhaustion scale**

**Items**	**Questionnaire Items**
**Emotional exhaustion**	Work makes me feel extremely exhausted both physically and mentally.
	I feel completely worn out when I get off work.
	When I get up in the morning and think about having to face a day’s work, I feel very tired.
	Working all day really puts a lot of pressure on me.
	Work makes me feel like I’m on the verge of a breakdown.

**Approach job crafting scale**

**Items**	**Questionnaire Items**
**Work organization**	I make my work process more systematic.
	I make my work environment more organized.
	I make my work plan more organized.
	I plan systematically and prioritize my work.
**Adaptability**	I use new knowledge or technologies to enhance work communication.
	I actively seek training opportunities or relevant courses on new technologies by myself.
	I find relevant training opportunities or courses on my own to improve my work.
	I use new knowledge or technologies to automate my work and improve work efficiency.
	I use new knowledge and technologies to organize my work.
**Metacognition**	I find ways to keep myself in a good mood at work.
	I find ways to get rid of my bad mood at work.
	I will find ways to focus my energy on my work.
	I create a unique personal way of thinking to deal with my work.
	I find ways to prepare for future work.
**Role expansion**	I offer my opinions on important issues to enrich my work role.
	I expand my work content to safeguard my physical and mental state.
	I expand my work activities to obtain resources that are helpful for me to complete my work.
	I add work that can ensure the quality of delivery.
	I add elements that can improve safety to my work.
**Social expansion**	I actively interact with others at work.
	I actively improve the quality of communication with others at work.
	I actively develop my professional network at work.
	I actively strive to improve the quality of team interaction.

**Avoidance job crafting scale**

**Items**	**Questionnaire Items**
**Withdrawal**	I work in a way that allows me to avoid other people at work.
	I work in a way that enables me to avoid interacting with others while working.
	I work in a way that helps me avoid the tedious tasks at work.
**Role reduction**	I find ways to let others attend the meeting in my place.
	I find ways to hand over my work to others outside the team.
	I find ways to reduce the time I spend in meetings.
	I find ways to avoid time-consuming work tasks.

**Idiosyncratic deals scale**

**Items**	**Questionnaire Items**
**Task customization**	Due to my excellent application of skills at work, I have successfully obtained more responsibilities that allow me to fully utilize those skills.
	At my request, my supervisor assigned me tasks that can better develop my skills.
	Through negotiations with my supervisor, my work tasks are now more in line with my personality, skills, and abilities.
	My supervisor provides me with opportunities to take on responsibilities beyond the formal job requirements (which I desire).
	In view of my unique contributions, my supervisor gives me more flexibility to freely choose the ways and means to complete my work.
	After my initial appointment, my supervisor arranged an ideal position for me where I can fully display my talents.
**Schedule flexibility**	My supervisor takes my personal needs into account when arranging the work schedule.
	At my request, my supervisor considers my needs for off-work leave and compensatory rest when allocating my working hours.
	Apart from business trips and sick leave, my supervisor allows me to take leave to handle matters outside of work.
**Location flexibility**	I negotiated a special arrangement with my supervisor that allows me to complete part of my work outside the office to meet my personal needs.
	Under special circumstances, my supervisor allows me to complete my work outside the workplace.
**Compensation incentives**	My supervisor ensures that my salary level can meet my personal needs.
	The company is willing to formulate a salary plan based on my actual abilities and performance.
	In view of my unique skills and contributions, my supervisor is willing to negotiate my salary with me.
	When I make additional contributions to the company, my supervisor will provide me with additional rewards outside the formal system.
	After my initial appointment, I negotiated with my supervisor to formulate a salary plan to reward my special contributions.
